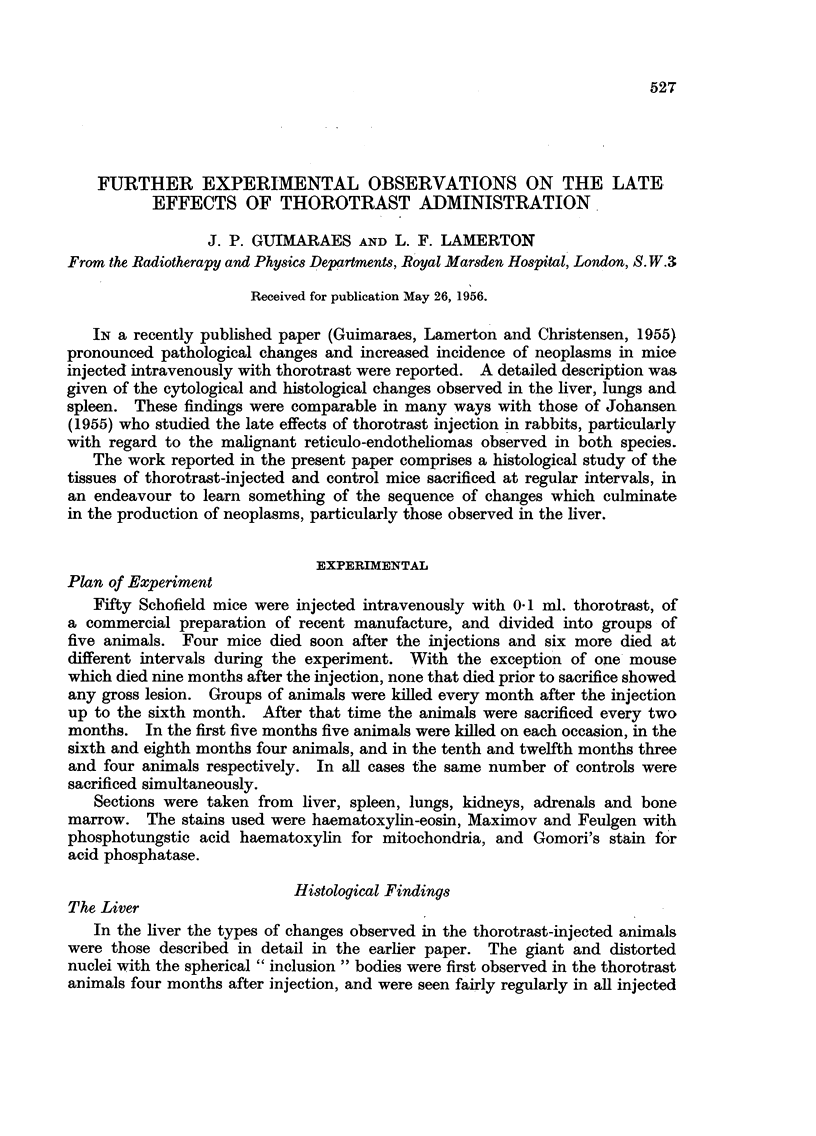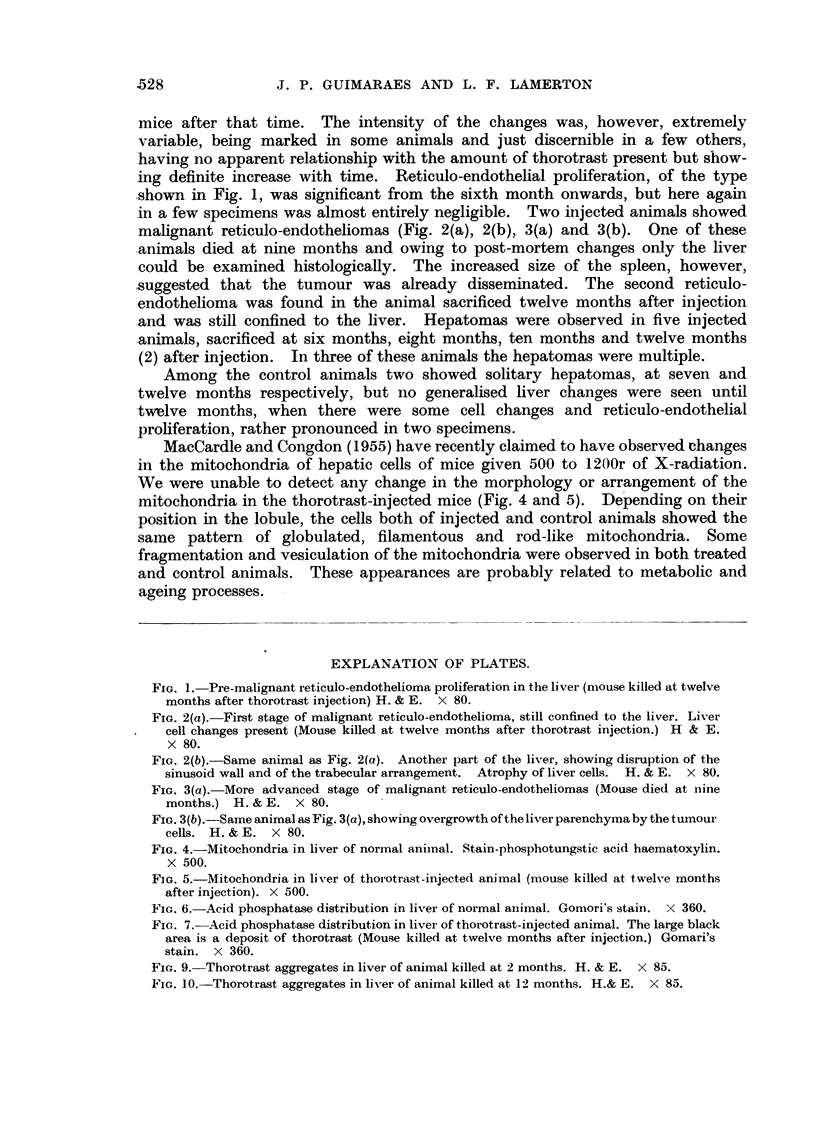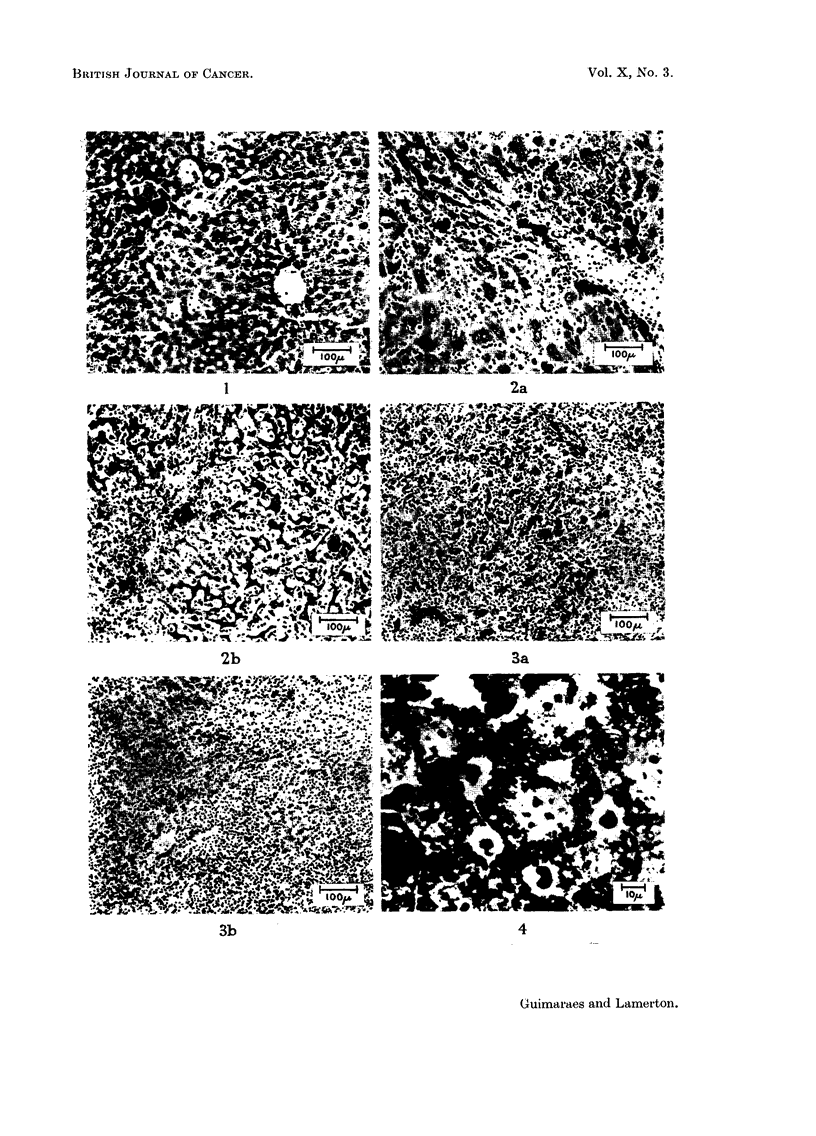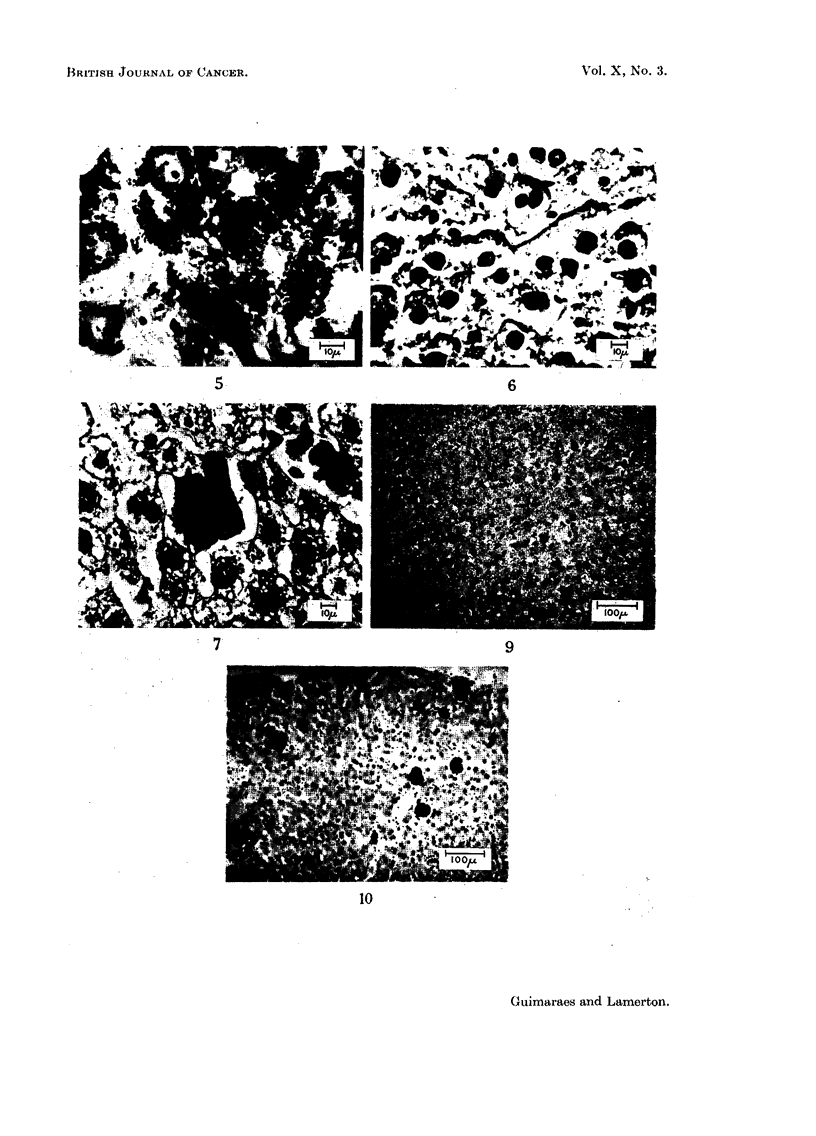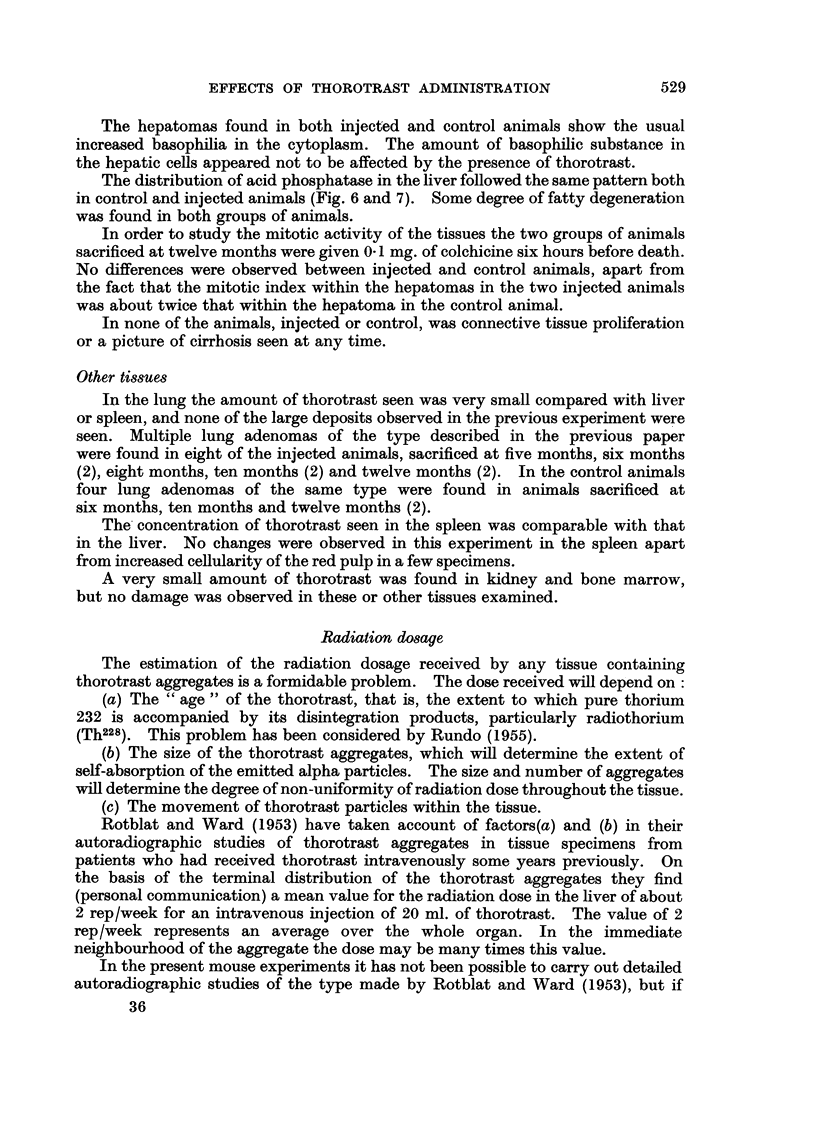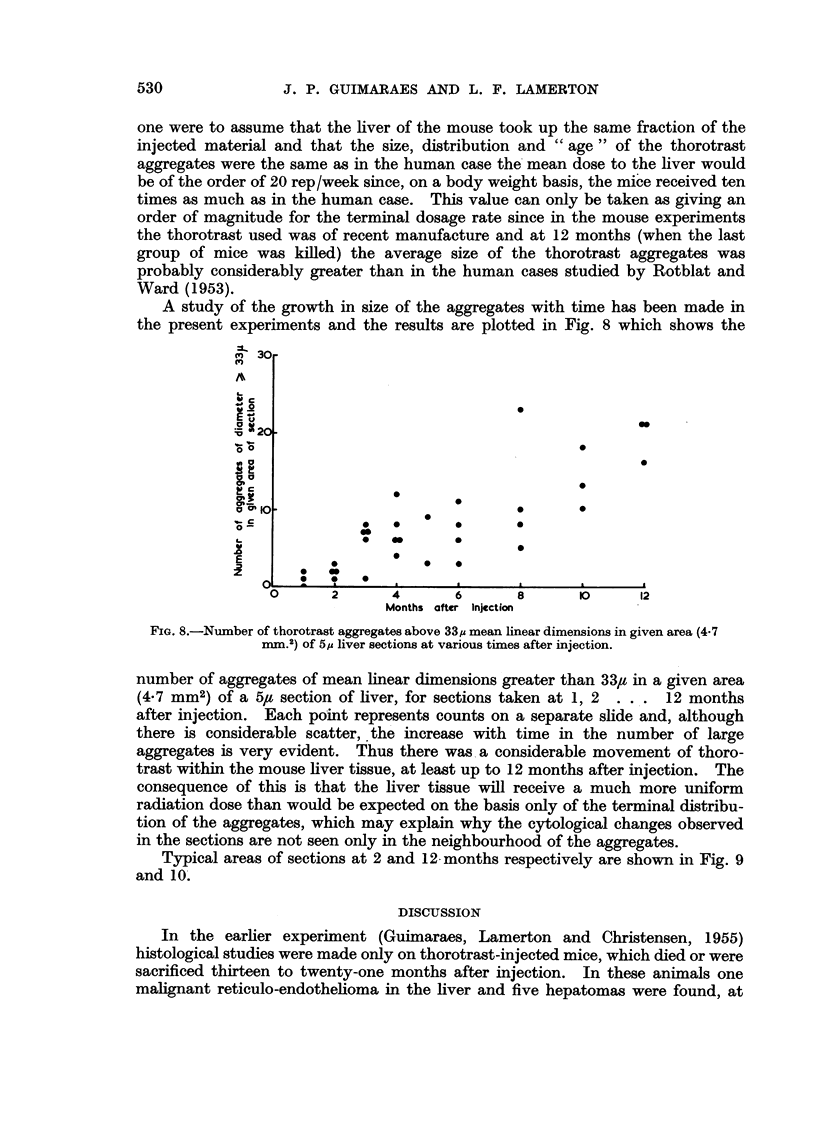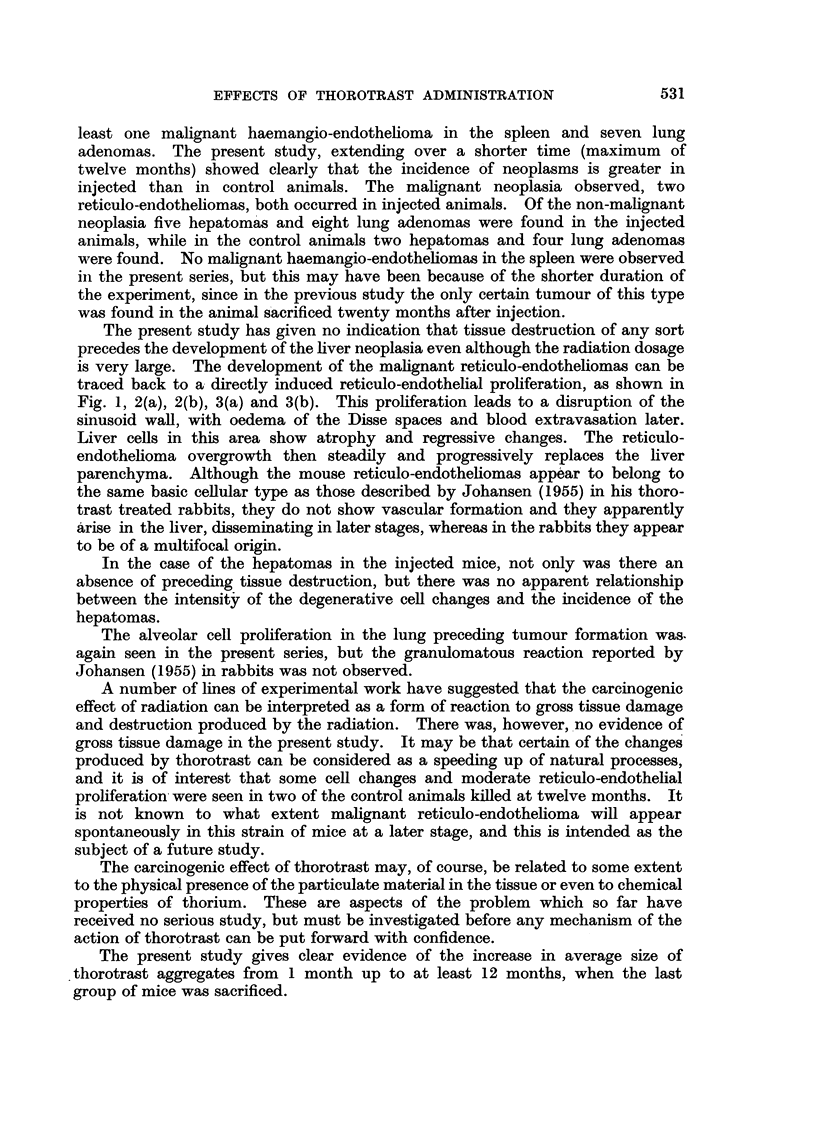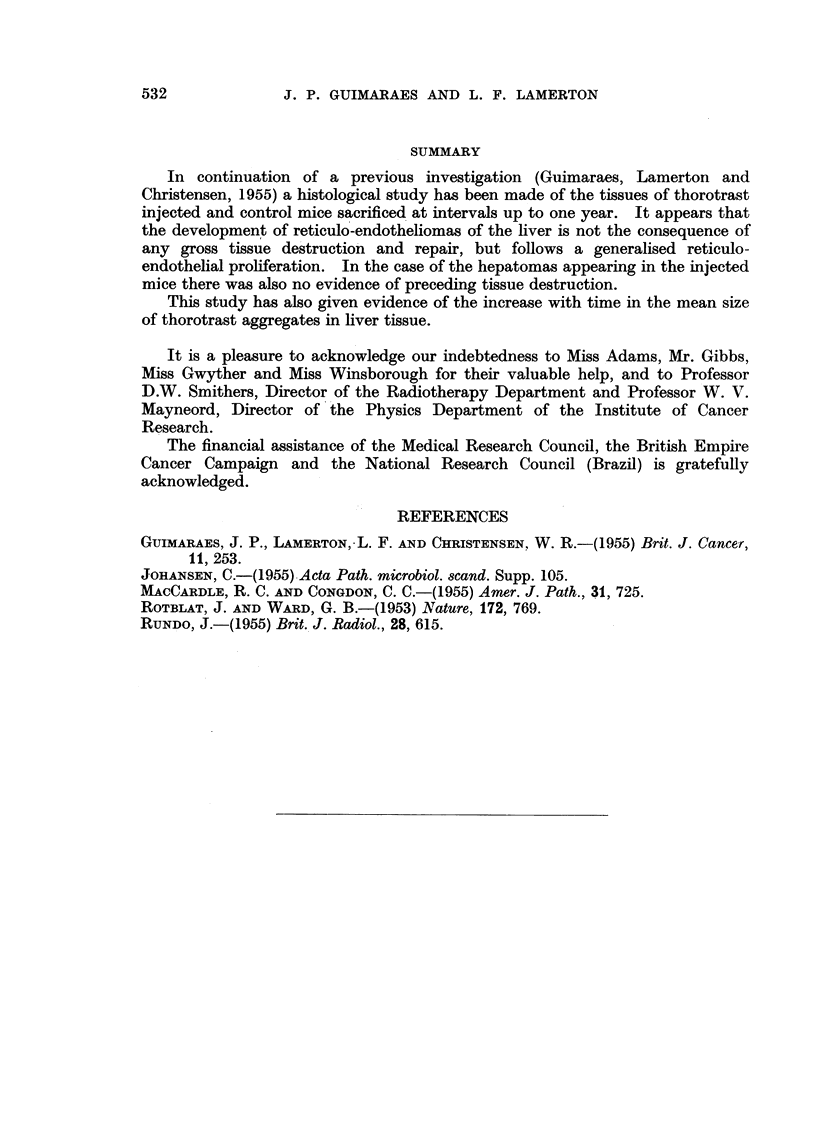# Further Experimental Observations on the Late Effects of Thorotrast Administration

**DOI:** 10.1038/bjc.1956.61

**Published:** 1956-09

**Authors:** J. P. Guimaraes, L. F. Lamerton

## Abstract

**Images:**


### FURTHER EXPERIMENTAL OBSERVATIONS ON THE LATE EFFECTS OF THOROTRAST ADMINISTRATION

J. P. GUIMARAES AND L. F. LAMERTON

*From the Radiotherapy and Physics Departments, Royal Marsden Hospital, London, S.W.3*

Received for publication May 26, 1956.

IN a recently published paper (Guimaraes, Lamerton and Christensen, 1955) pronounced pathological changes and increased incidence of neoplasms in mice injected intravenously with thorotrast were reported. A detailed description was given of the cytological and histological changes observed in the liver, lungs and spleen. These findings were comparable in many ways with those of Johansen (1955) who studied the late effects of thorotrast injection in rabbits, particularly with regard to the malignant reticulo-endotheliomas observed in both species.

The work reported in the present paper comprises a histological study of the tissues of thorotrast-injected and control mice sacrificed at regular intervals, in an endeavour to learn something of the sequence of changes which culminate in the production of neoplasms, particularly those observed in the liver.

### EXPERIMENTAL

### *Plan of Experiment*

Fifty Schofield mice were injected intravenously with 0·1 ml. thorotrast, of a commercial preparation of recent manufacture, and divided into groups of five animals. Four mice died soon after the injections and six more died at different intervals during the experiment. With the exception of one mouse which died nine months after the injection, none that died prior to sacrifice showed any gross lesion. Groups of animals were killed every month after the injection up to the sixth month. After that time the animals were sacrificed every two months. In the first five months five animals were killed on each occasion, in the sixth and eighth months four animals, and in the tenth and twelfth months three and four animals respectively. In all cases the same number of controls were sacrificed simultaneously.

Sections were taken from liver, spleen, lungs, kidneys, adrenals and bone marrow. The stains used were haematoxylin-eosin, Maximov and Feulgen with phosphotungstic acid haematoxylin for mitochondria, and Gomori's stain for acid phosphatase.

### *Histological Findings*

#### *The Liver*

In the liver the types of changes observed in the thorotrast-injected animals were those described in detail in the earlier paper. The giant and distorted nuclei with the spherical " inclusion " bodies were first observed in the thorotrast animals four months after injection, and were seen fairly regularly in all injected mice after that time. The intensity of the changes was, however, extremely variable, being marked in some animals and just discernible in a few others, having no apparent relationship with the amount of thorotrast present but showing definite increase with time. Reticulo-endothelial proliferation, of the type shown in Fig. 1, was significant from the sixth month onwards, but here again in a few specimens was almost entirely negligible. Two injected animals showed malignant reticulo-endotheliomas (Fig. 2(a), 2(b), 3(a) and 3(b). One of these animals died at nine months and owing to post-mortem changes only the liver could be examined histologically. The increased size of the spleen, however, suggested that the tumour was already disseminated. The second reticulo-endothelioma was found in the animal sacrificed twelve months after injection and was still confined to the liver. Hepatomas were observed in five injected animals, sacrificed at six months, eight months, ten months and twelve months (2) after injection. In three of these animals the hepatomas were multiple.

Among the control animals two showed solitary hepatomas, at seven and twelve months respectively, but no generalised liver changes were seen until twelve months, when there were some cell changes and reticulo-endothelial proliferation, rather pronounced in two specimens.

MacCardle and Congdon (1955) have recently claimed to have observed changes in the mitochondria of hepatic cells of mice given 500 to 1200r of X-radiation. We were unable to detect any change in the morphology or arrangement of the mitochondria in the thorotrast-injected mice (Fig. 4 and 5). Depending on their position in the lobule, the cells both of injected and control animals showed the same pattern of globulated, filamentous and rod-like mitochondria. Some fragmentation and vesiculation of the mitochondria were observed in both treated and control animals. These appearances are probably related to metabolic and ageing processes.

---

### EXPLANATION OF PLATES.

FIG. 1.—Pre-malignant reticulo-endothelioma proliferation in the liver (mouse killed at twelve months after thorotrast injection) H. & E. × 80.

FIG. 2(*a*).—First stage of malignant reticulo-endothelioma, still confined to the liver. Liver cell changes present (Mouse killed at twelve months after thorotrast injection.) H & E. × 80.

FIG. 2(*b*).—Same animal as Fig. 2(*a*). Another part of the liver, showing disruption of the sinusoid wall and of the trabecular arrangement. Atrophy of liver cells. H. & E. × 80.

FIG. 3(*a*).—More advanced stage of malignant reticulo-endotheliomas (Mouse died at nine months.) H. & E. × 80.

FIG. 3(*b*).—Same animal as Fig. 3(*a*), showing overgrowth of the liver parenchyma by the tumour cells. H. & E. × 80.

FIG. 4.—Mitochondria in liver of normal animal. Stain-phosphotungstic acid haematoxylin. × 500.

FIG. 5.—Mitochondria in liver of thorotrast-injected animal (mouse killed at twelve months after injection). × 500.

FIG. 6.—Acid phosphatase distribution in liver of normal animal. Gomori's stain. × 360.

FIG. 7.—Acid phosphatase distribution in liver of thorotrast-injected animal. The large black area is a deposit of thorotrast (Mouse killed at twelve months after injection.) Gomari's stain. × 360.

FIG. 9.—Thorotrast aggregates in liver of animal killed at 2 months. H. & E. × 85.

FIG. 10.—Thorotrast aggregates in liver of animal killed at 12 months. H.& E. × 85.

1

2a

2b

3a

3b

4

5

6

7

9

10

Guimaraes and Lamerton.

The hepatomas found in both injected and control animals show the usual increased basophilia in the cytoplasm. The amount of basophilic substance in the hepatic cells appeared not to be affected by the presence of thorotrast.

The distribution of acid phosphatase in the liver followed the same pattern both in control and injected animals (Fig. 6 and 7). Some degree of fatty degeneration was found in both groups of animals.

In order to study the mitotic activity of the tissues the two groups of animals sacrificed at twelve months were given 0·1 mg. of colchicine six hours before death. No differences were observed between injected and control animals, apart from the fact that the mitotic index within the hepatomas in the two injected animals was about twice that within the hepatoma in the control animal.

In none of the animals, injected or control, was connective tissue proliferation or a picture of cirrhosis seen at any time.

*Other tissues*

In the lung the amount of thorotrast seen was very small compared with liver or spleen, and none of the large deposits observed in the previous experiment were seen. Multiple lung adenomas of the type described in the previous paper were found in eight of the injected animals, sacrificed at five months, six months (2), eight months, ten months (2) and twelve months (2). In the control animals four lung adenomas of the same type were found in animals sacrificed at six months, ten months and twelve months (2).

The concentration of thorotrast seen in the spleen was comparable with that in the liver. No changes were observed in this experiment in the spleen apart from increased cellularity of the red pulp in a few specimens.

A very small amount of thorotrast was found in kidney and bone marrow, but no damage was observed in these or other tissues examined.

### *Radiation dosage*

The estimation of the radiation dosage received by any tissue containing thorotrast aggregates is a formidable problem. The dose received will depend on :

(*a*) The " age " of the thorotrast, that is, the extent to which pure thorium 232 is accompanied by its disintegration products, particularly radiothorium ($Th^{228}$). This problem has been considered by Rundo (1955).

(*b*) The size of the thorotrast aggregates, which will determine the extent of self-absorption of the emitted alpha particles. The size and number of aggregates will determine the degree of non-uniformity of radiation dose throughout the tissue.

(*c*) The movement of thorotrast particles within the tissue.

Rotblat and Ward (1953) have taken account of factors(*a*) and (*b*) in their autoradiographic studies of thorotrast aggregates in tissue specimens from patients who had received thorotrast intravenously some years previously. On the basis of the terminal distribution of the thorotrast aggregates they find (personal communication) a mean value for the radiation dose in the liver of about 2 rep/week for an intravenous injection of 20 ml. of thorotrast. The value of 2 rep/week represents an average over the whole organ. In the immediate neighbourhood of the aggregate the dose may be many times this value.

In the present mouse experiments it has not been possible to carry out detailed autoradiographic studies of the type made by Rotblat and Ward (1953), but if one were to assume that the liver of the mouse took up the same fraction of the injected material and that the size, distribution and " age " of the thorotrast aggregates were the same as in the human case the mean dose to the liver would be of the order of 20 rep/week since, on a body weight basis, the mice received ten times as much as in the human case. This value can only be taken as giving an order of magnitude for the terminal dosage rate since in the mouse experiments the thorotrast used was of recent manufacture and at 12 months (when the last group of mice was killed) the average size of the thorotrast aggregates was probably considerably greater than in the human cases studied by Rotblat and Ward (1953).

A study of the growth in size of the aggregates with time has been made in the present experiments and the results are plotted in Fig. 8 which shows the

FIG. 8.—Number of thorotrast aggregates above 33$\mu$ mean linear dimensions in given area (4·7 mm.$^2$) of 5$\mu$ liver sections at various times after injection.

number of aggregates of mean linear dimensions greater than 33$\mu$ in a given area (4·7 mm$^2$) of a 5$\mu$ section of liver, for sections taken at 1, 2 . . . 12 months after injection. Each point represents counts on a separate slide and, although there is considerable scatter, the increase with time in the number of large aggregates is very evident. Thus there was a considerable movement of thorotrast within the mouse liver tissue, at least up to 12 months after injection. The consequence of this is that the liver tissue will receive a much more uniform radiation dose than would be expected on the basis only of the terminal distribution of the aggregates, which may explain why the cytological changes observed in the sections are not seen only in the neighbourhood of the aggregates.

Typical areas of sections at 2 and 12 months respectively are shown in Fig. 9 and 10.

### DISCUSSION

In the earlier experiment (Guimaraes, Lamerton and Christensen, 1955) histological studies were made only on thorotrast-injected mice, which died or were sacrificed thirteen to twenty-one months after injection. In these animals one malignant reticulo-endothelioma in the liver and five hepatomas were found, at least one malignant haemangio-endothelioma in the spleen and seven lung adenomas. The present study, extending over a shorter time (maximum of twelve months) showed clearly that the incidence of neoplasms is greater in injected than in control animals. The malignant neoplasia observed, two reticulo-endotheliomas, both occurred in injected animals. Of the non-malignant neoplasia five hepatomas and eight lung adenomas were found in the injected animals, while in the control animals two hepatomas and four lung adenomas were found. No malignant haemangio-endotheliomas in the spleen were observed in the present series, but this may have been because of the shorter duration of the experiment, since in the previous study the only certain tumour of this type was found in the animal sacrificed twenty months after injection.

The present study has given no indication that tissue destruction of any sort precedes the development of the liver neoplasia even although the radiation dosage is very large. The development of the malignant reticulo-endotheliomas can be traced back to a directly induced reticulo-endothelial proliferation, as shown in Fig. 1, 2(a), 2(b), 3(a) and 3(b). This proliferation leads to a disruption of the sinusoid wall, with oedema of the Disse spaces and blood extravasation later. Liver cells in this area show atrophy and regressive changes. The reticulo-endothelioma overgrowth then steadily and progressively replaces the liver parenchyma. Although the mouse reticulo-endotheliomas appear to belong to the same basic cellular type as those described by Johansen (1955) in his thorotrast treated rabbits, they do not show vascular formation and they apparently arise in the liver, disseminating in later stages, whereas in the rabbits they appear to be of a multifocal origin.

In the case of the hepatomas in the injected mice, not only was there an absence of preceding tissue destruction, but there was no apparent relationship between the intensity of the degenerative cell changes and the incidence of the hepatomas.

The alveolar cell proliferation in the lung preceding tumour formation was again seen in the present series, but the granulomatous reaction reported by Johansen (1955) in rabbits was not observed.

A number of lines of experimental work have suggested that the carcinogenic effect of radiation can be interpreted as a form of reaction to gross tissue damage and destruction produced by the radiation. There was, however, no evidence of gross tissue damage in the present study. It may be that certain of the changes produced by thorotrast can be considered as a speeding up of natural processes, and it is of interest that some cell changes and moderate reticulo-endothelial proliferation were seen in two of the control animals killed at twelve months. It is not known to what extent malignant reticulo-endothelioma will appear spontaneously in this strain of mice at a later stage, and this is intended as the subject of a future study.

The carcinogenic effect of thorotrast may, of course, be related to some extent to the physical presence of the particulate material in the tissue or even to chemical properties of thorium. These are aspects of the problem which so far have received no serious study, but must be investigated before any mechanism of the action of thorotrast can be put forward with confidence.

The present study gives clear evidence of the increase in average size of thorotrast aggregates from 1 month up to at least 12 months, when the last group of mice was sacrificed.

SUMMARY

In continuation of a previous investigation (Guimaraes, Lamerton and Christensen, 1955) a histological study has been made of the tissues of thorotrast injected and control mice sacrificed at intervals up to one year. It appears that the development of reticulo-endotheliomas of the liver is not the consequence of any gross tissue destruction and repair, but follows a generalised reticulo-endothelial proliferation. In the case of the hepatomas appearing in the injected mice there was also no evidence of preceding tissue destruction.

This study has also given evidence of the increase with time in the mean size of thorotrast aggregates in liver tissue.

It is a pleasure to acknowledge our indebtedness to Miss Adams, Mr. Gibbs, Miss Gwyther and Miss Winsborough for their valuable help, and to Professor D.W. Smithers, Director of the Radiotherapy Department and Professor W. V. Mayneord, Director of the Physics Department of the Institute of Cancer Research.

The financial assistance of the Medical Research Council, the British Empire Cancer Campaign and the National Research Council (Brazil) is gratefully acknowledged.

### REFERENCES

GUIMARAES, J. P., LAMERTON, L. F. AND CHRISTENSEN, W. R.—(1955) *Brit. J. Cancer*, **11**, 253.

JOHANSEN, C.—(1955) *Acta Path. microbiol. scand.* Supp. 105.

MACCARDLE, R. C. AND CONGDON, C. C.—(1955) *Amer. J. Path.*, **31**, 725.

ROTBLAT, J. AND WARD, G. B.—(1953) *Nature*, **172**, 769.

RUNDO, J.—(1955) *Brit. J. Radiol.*, **28**, 615.